# 高效液相色谱法测定氧化型染发产品中40种染发剂

**DOI:** 10.3724/SP.J.1123.2020.11020

**Published:** 2021-11-08

**Authors:** Xue ZUO, Zheng DI, Yong DU, Ling YANG, Rong ZHANG, Guoqing WU

**Affiliations:** 北京市药品检验所, 北京 102206; Beijing Institute for Drug Control, Beijing 102206, China; 北京市药品检验所, 北京 102206; Beijing Institute for Drug Control, Beijing 102206, China; 北京市药品检验所, 北京 102206; Beijing Institute for Drug Control, Beijing 102206, China; 北京市药品检验所, 北京 102206; Beijing Institute for Drug Control, Beijing 102206, China; 北京市药品检验所, 北京 102206; Beijing Institute for Drug Control, Beijing 102206, China; 北京市药品检验所, 北京 102206; Beijing Institute for Drug Control, Beijing 102206, China

**Keywords:** 高效液相色谱, 染发剂, 氧化型染发产品, high performance liquid chromatography (HPLC), dyes, oxidative hair dye products

## Abstract

氧化型染发产品中的多种染发剂具有不同程度的致敏性及其他毒性,建立快速、准确检测多种染发剂的方法,为该类产品监管提供有效的技术手段,十分必要。该研究建立了氧化型染发类产品中40种染发剂的高效液相色谱测定方法。染发产品经含70%乙醇的亚硫酸氢钠水溶液涡旋、超声提取,并经亚硫酸氢钠水溶液稀释后,以0.02 mol/L乙酸铵水溶液(含4%乙腈)和乙腈为流动相,采用Waters Atlantis^®^ T3 MV Kit色谱柱(250 mm×4.6 mm, 5 μm)分离,配合柱温变化进行梯度洗脱,二极管阵列检测器检测,检测波长为235 nm和280 nm,外标法定量。结果表明,40种染发剂在各自范围内线性关系良好,相关系数均大于0.999; 40种染发剂的检出限为5~168 μg/g,定量限为16~504 μg/g;各染发剂在3个添加水平下的平均回收率为81.4%~109.6%, RSD均小于5%;各染发剂标准溶液在24 h内稳定性良好,RSD为0.2%~2.2%。与现行标准检验方法相比,该方法较大程度地增加了单一液相色谱条件下可测定的染发剂,特别是准用染发剂种类(36种),提高了检测效率,并可保证检测结果的灵敏度与准确性,适用于氧化型染发产品中多种染发剂的检测分析。

## Abstract (en)

Oxidative hair dye products have always been one of the key concerns in cosmetic supervision. The various aromatic amine and phenol dyes used in such products have different degrees of sensitization and toxicities. Therefore, it is necessary to establish a rapid and accurate method for the determination of various dyes. A high performance liquid chromatography (HPLC) method was developed for the determination of 40 dyes in oxidative hair dye products, and the method was optimized in terms of mobile phase type, column temperature, detection wavelength, and extraction solvent concentration. The sample pretreatment method was as follows: weighed 0.5 g sample accurately, added to 10 mL of sodium bisulfite solution containing 70% ethanol, extracted by ultrasonication for 15 min, added to 25 mL of the sodium bisulfite solution. Chromatographic separation was carried out on a Waters Atlantis^®^ T3 MV Kit column (250 mm×4.6 mm, 5 μm) by gradient elution. To protect the column, the sample solution was filtered using a syringe filter with a 0.45-μm membrane before injection. The mobile phase was 0.02 mol/L ammonium acetate aqueous solution (containing 4% acetonitrile) and acetonitrile. The column temperature was varied between 30 ℃ and 35 ℃ during the separation. The dyes were detected using a photodiode array detector. The detection wavelength of 31 dyes was 235 nm, and that of the other nine dyes was 280 nm. The external standard method was used for quantitative analysis. The results showed that the 40 dyes could be separated well. All the dyes had good linearities in their own concentration ranges, with correlation coefficients (*r*) exceeding 0.999. The limits of detection (LODs) of the 40 dyes ranged from 5 to 168 μg/g, and the limits of quantification (LOQs) ranged from 16 to 504 μg/g. The average spiked recoveries at three levels (2.5, 5.0, and 10 mg/g) of the 40 dyes ranged from 81.4% to 109.6%, with relative standard deviations (RSDs) less than 5%. The stability of each dye in the standard solutions was good within 24 h, with RSDs ranging from 0.2% to 2.2%. To demonstrate the applicability of the method, 12 batches of commercial oxidative hair dye products from different manufacturers were analyzed. In total, 24 kinds of dyes were detected in the 12 batches of samples, and the contents of these dyes ranged from 0.01% to 2.83%. The method greatly increased the types of dyes detected by a single chromatography step, especially the permitted dyes (36 types). Compared with the standard method of Safety and Technical Standards for Cosmetics (2015 edition), this method replenished 13 permitted dyes: 1-hydroxyethyl 4,5-diaminopyrazole sulfate, hydroxyethyl-*p*-phenylenediaminesulfate, tetraaminopyrimidine sulfate, 2,6-dihydroxyethylaminotoluene, 2-amino-6-chloro-4-nitrophenol, 2-methyl-5-hydroxyethylaminophenol, 3-nitro-*p*-hydroxyethylaminophenol, 4-hydroxypropylamino-3-nitrophenol, 5-amino-4-chloro-*o*-cresol, 5-amino-6-chloro-*o*-cresol, HC yellow No. 2, hydroxybenzomorpholine, and hydroxyethyl-2-nitro-*p*-toluidine. The sensitivity and accuracy of the results could ensure and the detection efficiency could be improved through validation of the method. The developed method is suitable for the determination of various dyes in oxidative hair dye products and can provide an effective technical means for the supervision of such products.

随着居民生活水平的日益提升,我国染发类产品的市场需求也日趋增加。染发产品按作用机理可分为氧化型和非氧化型,其中氧化型为目前市场上销售、使用的主要产品类型。氧化型染发产品中的染发剂主要包括芳香胺类及酚类化合物等,相关研究表明,此类化合物具有不同程度的致敏性、致突变性及其他毒性作用^[1,2,3,4]^,可引发皮肤过敏等不良反应^[5,6,7]^,使该类产品的安全性备受关注。因此,针对氧化型染发产品中常见的染发剂,建立快速、准确的检测方法,为产品的研发与监管提供有效的技术手段,是十分必要的。

目前,包括液相色谱法^[8,9,10,11,12,13]^、液相色谱-质谱法^[14,15,16,17,18]^、气相色谱法^[19]^、气相色谱-质谱法^[20,21]^、离子色谱法^[22]^及毛细管电泳法^[23]^等在内的多种技术手段均已应用于染发剂的检测。其中,液相色谱法、液相色谱-质谱法应用最为广泛,但质谱仪器成本较高,且在分析复杂样品及成分含量未知的样品时,易造成离子源污染;液相色谱法适用于多种染发剂的分离分析,是一种比较理想的检验方法。查询、统计国家药品监督管理局2016~2018年注册的染发产品配方,并结合文献^[24]^报道,发现目前氧化型染发产品中添加的准用染发剂达四十余种。《化妆品安全技术规范》(2015年版,简称《规范》)^[25]^中提供的对苯二胺等32种组分的法定检验方法及严巍等^[26]^建立的32种禁限用染发剂的液相色谱测定方法,均可实现32种禁用、准用染发剂(其中包含24种准用染发剂)的检测,但需要同时建立3个液相色谱系统才能完成测定,仪器、色谱柱及试剂的消耗很大,且影响检验效率。近几年,多篇研究^[11,27-29]^报道了采用单一液相色谱体系同时测定多种染发剂的检测方法,方法较为简便、高效,但最多可检测不到30种染发剂。

本研究利用单一液相色谱系统建立了40种染发剂的定性、定量测定方法,其中包含36种准用染发剂。与《规范》^[25]^相比,该方法大大简化了液相色谱分析条件,并增加了1-羟乙基-4,5-二氨基吡唑硫酸盐、羟乙基对苯二胺硫酸盐、四氨基嘧啶硫酸盐、2,6-二羟乙基氨甲苯、2-氨基-6-氯-4-硝基苯酚、2-甲基-5-羟乙氨基苯酚、3-硝基对羟乙氨基酚、4-羟丙氨基-3-硝基苯酚、5-氨基-4-氯邻甲酚、5-氨基-6-氯-邻甲酚、HC黄2号、羟苯并吗啉和羟乙基-2-硝基对甲苯胺等13种常见准用染发剂,较大程度扩充了染发剂检测种类。与实验室前期建立的、同时测定33种染发剂的液相色谱方法^[30]^相比,该方法通过改变流动相种类、增加柱温变化等方式,进一步提升了各染发剂的分离效果,弥补了1-羟乙基-4,5-二氨基吡唑硫酸盐、四氨基嘧啶硫酸盐、2-氨基-3-羟基吡啶和对苯二胺等大极性组分分离度不佳的问题;该方法补充了《规范》^[25]^方法含有、而实验室前期建立方法中未包含的准用染发剂苯基甲基吡唑啉酮,同时还增加了《规范》^[25]^与前期方法中均未包含的2,6-二羟乙基氨甲苯等10种准用染发剂,实现了更多成分的有效分离与准确定量,提升了方法在产品实际检验工作中的全面性与针对性;方法通过提取溶剂浓度优化,进一步提高了样品提取率与检测结果准确性。该方法为氧化型染发产品的高效、经济、准确检测以及更全面监管提供了技术支持。

## 1 实验部分

### 1.1 仪器和试剂

1100型高效液相色谱仪配二极管阵列检测器(DAD),美国Agilent公司;CP225D型电子分析天平,德国Sartorius公司;KQ-500型超声波清洗器,昆山市超声仪器有限公司。

1,5-萘二酚等14种染发剂对照品购自北京曼哈格生物科技有限公司,1-萘酚等13种染发剂对照品购自德国Dr. Ehrenstorfer公司,2-氨基-6-氯-4-硝基苯酚等6种染发剂对照购自美国Sinco Pharmachem公司,2-甲基-5-羟乙氨基苯酚等3种染发剂对照品购自加拿大Toronto Research Chemicals公司,1-羟乙基-4,5-二氨基吡唑硫酸盐等3种染发剂对照品购自美国Ark Pharm公司,四氨基嘧啶硫酸盐购自北京百灵威科技有限公司。所有对照品纯度均不低于95%。40种染发剂对照品信息详见表1。

**表1:** 40种染发剂对照品的信息

No.	t_R_/min	Compound
1	3.550	1-hydroxyethyl 4,5-diaminopyrazole sulfate (1-羟乙基-4,5-二氨基吡唑硫酸盐)
2	3.894	tetraaminopyrimidine sulfate (四氨基嘧啶硫酸盐)
3	5.472	2-amino-3-hydroxypyridine (2-氨基-3-羟基吡啶)
4	5.910	p-phenylenediamine (对苯二胺)
5	6.608	hydroxyethyl-p-phenylenediaminesulfate (羟乙基对苯二胺硫酸盐)
6	7.650	p-aminophenol (对氨基苯酚)
7	9.410	2,6-diaminopyridine (2,6-二氨基吡啶)
8	9.945	m-phenylenediamine (间苯二胺)
9	10.280	hydroquinone (氢醌)
10	11.035	toluene-2,5-diamine sulfate (甲苯-2,5-二胺硫酸盐)
11	12.433	2,4-diaminophenoxyethanol HCl (2,4-二氨基苯氧基乙醇盐酸盐)
12	13.198	m-aminophenol (间氨基苯酚)
13	14.916	4-amino-m-cresol (4-氨基间甲酚)
14	17.741	o-phenylenediamine (邻苯二胺)
15	19.567	resorcinol (间苯二酚)
16	20.352	o-aminophenol (邻氨基苯酚)
17	22.257	2-chloro-p-phenylenediamine sulfate (2-氯-对苯二胺硫酸盐)
18	23.063	N,N-bis(2-hydroxyethyl)-p-phenylenediamine sulfate (N,N-双(2-羟乙基)-对苯二胺硫酸盐)
19	24.357	2-methylresorcinol (2-甲基间苯二酚)
20	29.024	2-amino-6-chloro-4-nitrophenol (2-氨基-6-氯-4-硝基苯酚)
21	34.799	hydroxybenzomorpholine (羟苯并吗啉)
22	38.056	4-amino-2-hydroxytoluene (4-氨基-2-羟基甲苯)
23	38.803	4-nitro-o-phenylenediamine (4-硝基邻苯二胺)
24	39.508	4-amino-3-nitrophenol (4-氨基-3-硝基苯酚)
25	41.934	6-amino-m-cresol (6-氨基间甲酚)
26	43.979	2,6-dihydroxyethylaminotoluene (2,6-二羟乙基氨甲苯)
27	45.960	3-nitro-p-hydroxyethylaminophenol (3-硝基对羟乙氨基酚)
28	46.662	2-methyl-5-hydroxyethylaminophenol (2-甲基-5-羟乙氨基苯酚)
29	47.630	pyrazolone methyl pyrazolone (苯基甲基吡唑啉酮)
30	50.582	4-chlororesorcinol (4-氯间苯二酚)
31	51.254	6-hydroxyindole (6-羟基吲哚)
32	52.596	5-amino-6-chloro-o-cresol (5-氨基-6-氯-邻甲酚)
33	53.513	4-hydroxypropylamino-3-nitrophenol (4-羟丙氨基-3-硝基苯酚)
34	55.264	1,5-naphthalenediol (1,5-萘二酚)
35	58.122	2,7-naphthalenediol (2,7-萘二酚)
36	60.632	HC yellow No.2 (HC黄2号)
37	61.270	5-amino-4-chloro-o-cresol (5-氨基-4-氯邻甲酚)
38	62.577	hydroxyethyl-2-nitro-p-toluidine (羟乙基-2-硝基对甲苯胺)
39	63.169	N-phenyl-p-phenylenediamine (N-苯基-对苯二胺)
40	63.735	1-naphthol (1-萘酚)

乙腈(色谱纯)购自德国Merck公司;Milli-Q超纯水系统购自法国Millipore公司;乙酸铵(色谱纯)购自美国Fisher Chemical公司;无水乙醇(优级纯)购自现代东方(北京)科技发展有限公司;亚硫酸氢钠(分析纯)购自北京化学试剂公司。

样品均为市售氧化型染发产品,含染发膏和氧化乳两种剂型,取其中染发膏供含量测定。

### 1.2 溶液配制

含70%乙醇的亚硫酸氢钠水溶液:量取无水乙醇700 mL,置于1000 mL量瓶中,加入2 g/L亚硫酸氢钠水溶液至刻度,充分振摇混匀,备用。

标准储备溶液:称取40种染发剂对照品各50 mg(精确至0.1 mg),置于同一50 mL棕色量瓶中,加入约40 mL含70%乙醇的亚硫酸氢钠水溶液,超声至充分溶解,再加入2 g/L亚硫酸氢钠水溶液至刻度,充分振摇混匀,即得混合标准储备溶液。

标准系列溶液:上述40种染发剂混合标准储备溶液用2g/L亚硫酸氢钠水溶液配制成系列标准溶液,质量浓度分别为5、10、50、100、250和500 mg/L。所得溶液于5 ℃条件下保存。

### 1.3 样品前处理

称取染发产品中染发膏0.5 g(精确至0.001 g),置于25 mL比色管中,加入含70%乙醇的亚硫酸氢钠水溶液至10 mL刻度,涡旋30~60 s至分散均匀,超声提取15 min,放冷后加入2 g/L亚硫酸氢钠水溶液至25 mL刻度,涡旋30 s,充分振摇混匀。经0.45 μm微孔滤膜过滤,续滤液待测。

### 1.4 色谱条件

色谱柱为Waters Atlantis^®^ T3 MV Kit柱(250 mm×4.6 mm, 5 μm);流动相:A相为0.02 mol/L乙酸铵水溶液(含4%乙腈), B相为乙腈;流速为1.0 mL/min;进样量为5 μL。柱温及梯度洗脱程序见表2。同时设置2个波长进行检测,四氨基嘧啶硫酸盐、间苯二酚、1,5-萘二酚、2,7-萘二酚、HC黄2号、5-氨基-4-氯邻甲酚、羟乙基-2-硝基对甲苯胺、*N*-苯基-对苯二胺、1-萘酚等9种染色剂的检测波长为280 nm,其余染色剂的检测波长均为235 nm。样品盘温度设置为5 ℃。

**表2:** HPLC梯度洗脱程序

Time/min	φ(A)/%	φ(B)/%	Column temperature/ ℃
0	100	0	30
25	100	0	30
25.1	100	0	35
30	95	5	35
38	90	10	35
47	87	13	35
55	75	25	35
60	35	65	35
61	100	0	30
67	100	0	30

A: 0.02 mol/L ammonium acetate aqueous solution (containing 4% acetonitrile); B: acetonitrile.

## 2 结果与讨论

### 2.1 流动相的选择

在实验室前期研究^[30]^中,选用磷酸盐缓冲体系(0.04 mol/L磷酸二氢钾和0.01 mol/L磷酸氢二钠,并含4%乙腈)和乙腈为流动相,通过梯度洗脱实现了33种染发剂的分离分析,但其中1-羟乙基-4,5-二氨基吡唑硫酸盐与四氨基嘧啶硫酸盐、2-氨基-3-羟基吡啶与对苯二胺、4-硝基邻苯二胺与4-氨基-3-硝基苯酚的分离效果有待提高。因此,本研究分别以上述磷酸盐缓冲体系-乙腈以及乙酸铵水溶液(含4%乙腈)-乙腈为流动相,比较两种体系对各种染发剂的分离效果。结果表明,乙酸铵体系(见图1a)对10 min内出峰染发剂的洗脱效果明显优于磷酸盐体系(见图1b),可有效解决极性较大染发剂的分离问题;两个体系(见图1c和1d)对10 min后出峰染发剂的洗脱效果相当。综上,选用乙酸铵体系作为流动相。在此基础上,比较不同浓度(0.01、0.02和0.03 mol/L)乙酸铵水溶液的分离效果,发现0.02 mol/L及0.03 mol/L乙酸铵水溶液的分离效果相当,均优于0.01 mol/L乙酸铵水溶液。从节约试剂及保护色谱柱的角度考虑,最终选择0.02 mol/L乙酸铵水溶液(含4%乙腈)和乙腈作为流动相。

**图1:**
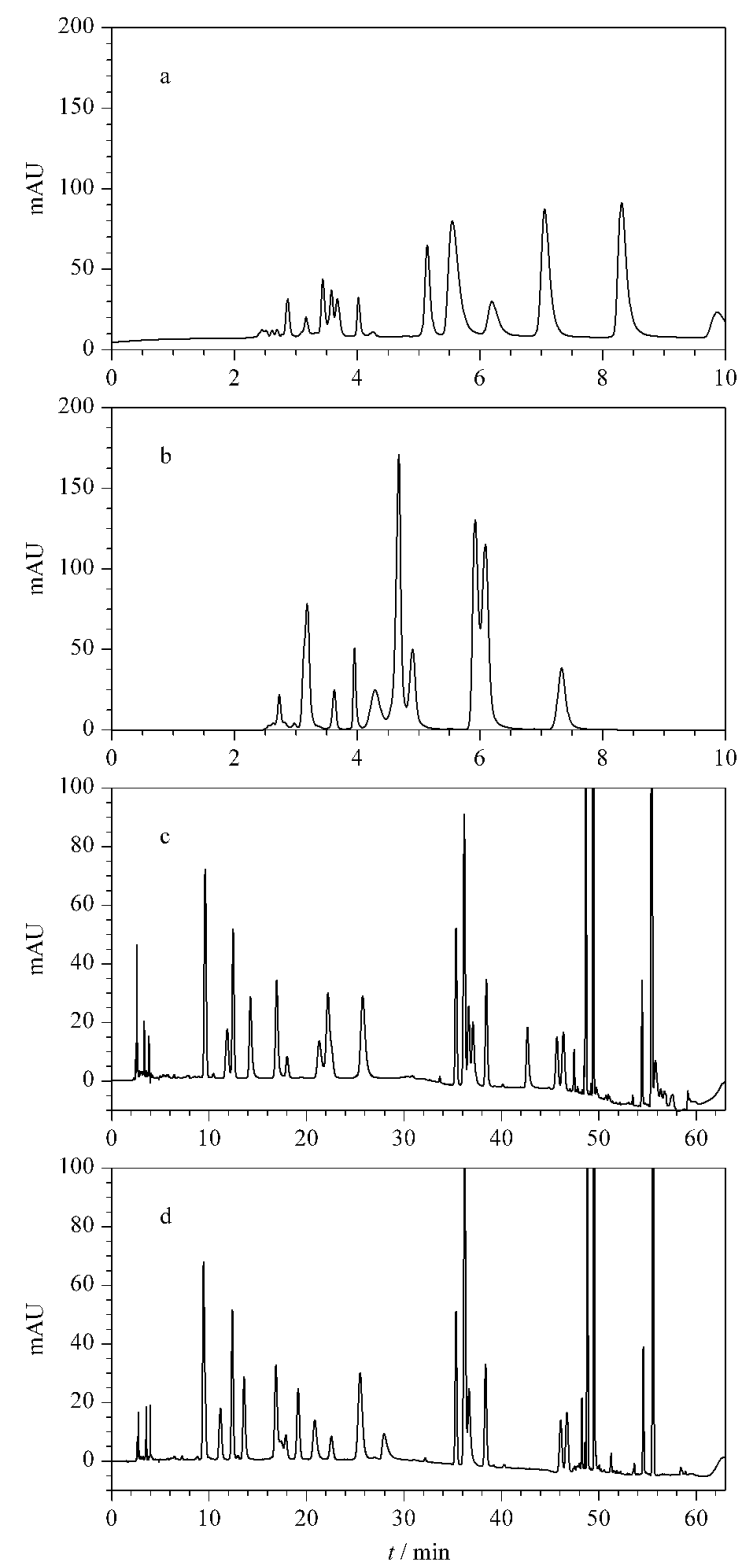
采用不同流动相时染发剂混合标准溶液的色谱图

### 2.2 色谱柱及柱温的选择

继续使用前期研究^[30]^选定的Waters Atlantis^®^ T3 MV Kit色谱柱(250 mm×4.6 mm, 5 μm)进行分离,并比较25、30和35 ℃柱温下的分离效果。结果表明,适当升温有助于色谱峰峰形的改善,可一定程度提高各染发剂的分离度。故进一步对30 ℃与35 ℃柱温条件进行比较,发现30 ℃柱温更利于25 min前出峰的染发剂的分离,而35 ℃更利于30 min后出峰的染发剂的分离。综上,考虑采用梯度变温的方式,25 min前柱温为30 ℃, 30 min后为35 ℃, 25~30 min色谱峰少,在此段时间内完成升温比较合适,故将此段时间作为变温过渡期。另外,为保证多次进样之间的重复性,在60 min后将温度降回初始温度30 ℃,并维持6 min,以实现系统平衡。经过多种尝试,最终确定1.4节描述的变温程序,确保绝大多数染发剂分离良好,且未见保留时间漂移。

### 2.3 检测波长的选择

采用DAD检测器在210~400 nm范围对40种染发剂进行全波长扫描,发现多数染发剂的最大吸收波长为220~245 nm,而235 nm下各染发剂均有较强的紫外吸收,认为该波长可以兼顾各成分的检测,因此首选235 nm为检测波长。但有9种染发剂存在特殊情况,具体如下:四氨基嘧啶硫酸盐、间苯二酚及*N*-苯基-对苯二胺在280 nm下吸收强于235 nm; 60 min后出峰的HC黄2号、5-氨基-4-氯邻甲酚、羟乙基-2-硝基对甲苯胺、*N*-苯基-对苯二胺及1-萘酚在235 nm下受到杂质峰干扰较严重;235 nm下,1,5-萘二酚、2,7-萘二酚及1-萘酚高浓度点响应过载。以上9种染发剂除了在低波长有最大吸收外,在275~310 nm有特征吸收,而280 nm可保证各成分的紫外吸收均较强,因此选择280 nm作为这9种染发剂的检测波长。利用1.4节色谱条件进行检测,得到40种染发剂在两种波长下的色谱图见图2。

**图2:**
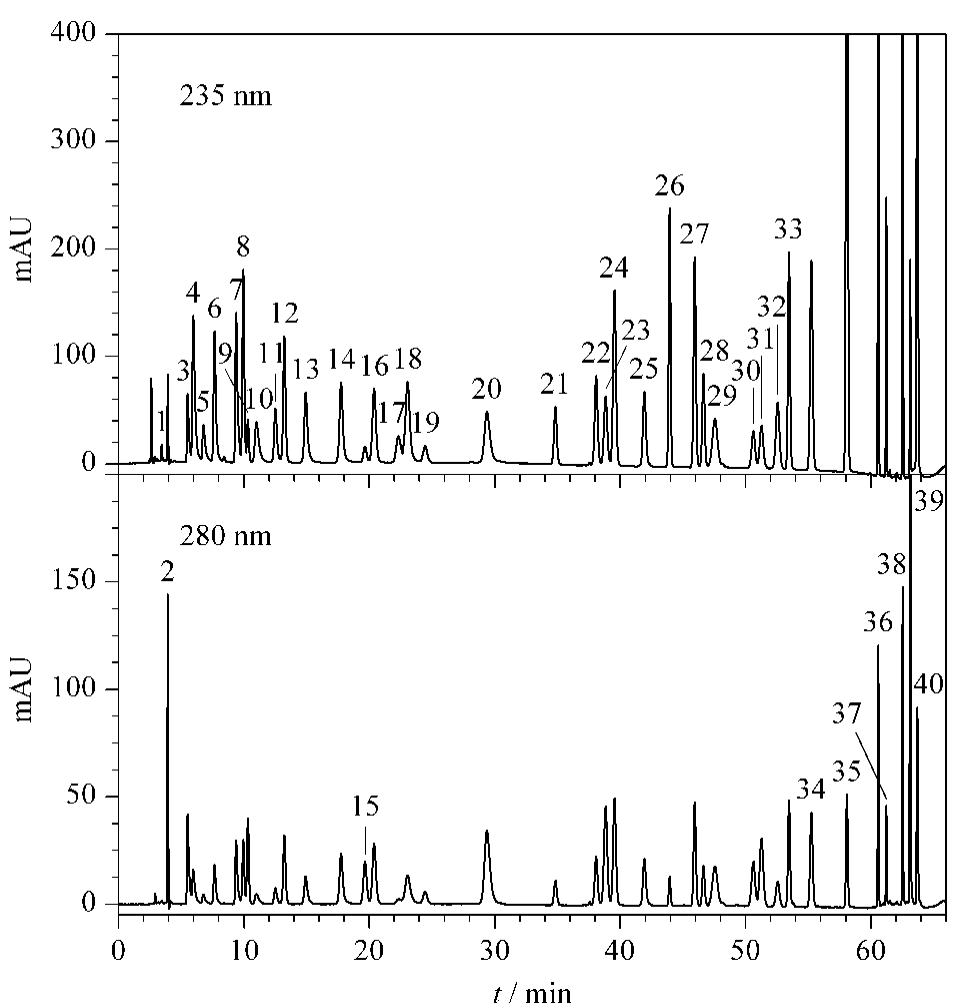
40种染发剂混合标准溶液在两种波长下的色谱图

### 2.4 方法适用性

在确定流动相与色谱柱种类后,对梯度洗脱条件进行摸索与优化,最终确定1.4节下的色谱条件,利用此条件进行分析,40种染发剂可实现有效分离(见图2)。

同时发现,采用本文方法检测准用染发剂对甲基氨基苯酚硫酸盐时,其与*N*,*N*-双(2-羟乙基)-对苯二胺硫酸盐保留时间非常接近。实际检验过程中,若样品只含这2种染发剂中的1种,则可通过紫外光谱确定染发剂种类(见图3);若样品中同时含有上述2种染发剂,则可参考实验室前期建立的33种染发剂的测定方法^[30]^,即采用25 ℃柱温,以磷酸盐缓冲液(0.04 mol/L磷酸二氢钾和0.01 mol/L磷酸氢二钠)-乙腈(96:4, v/v)作为流动相进行等度洗脱,该方法可实现2种染发剂的有效分离与准确定量。

**图3:**
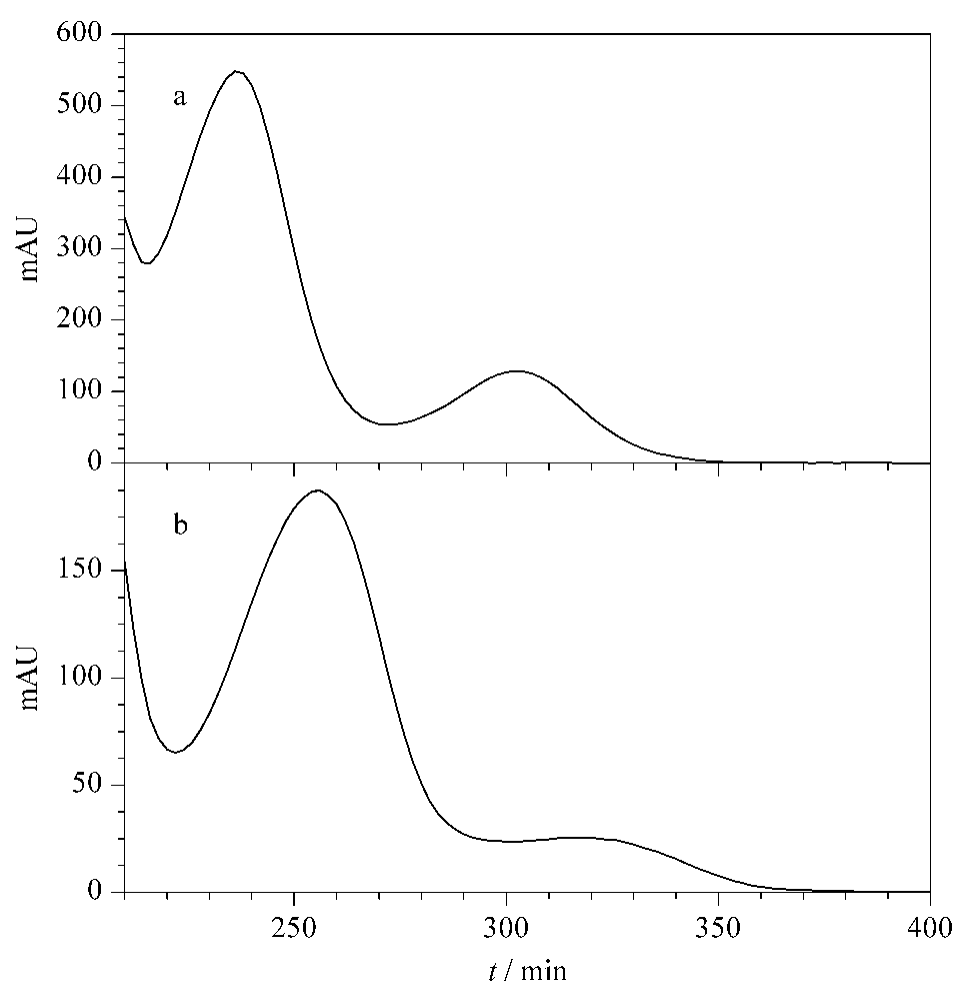
2种染发剂的紫外吸收光谱图

在国家药品监督管理局网站上查询2016~2018年注册的1000余种染发产品批件的配方信息。统计表明,3年注册的国产染发类产品中,未见对甲基氨基苯酚硫酸盐与*N*,*N*-双(2-羟乙基)-对苯二胺硫酸盐同时添加使用的情况;而仅7种进口产品同时添加了上述2种染发剂。可见,这2种染发剂同时添加使用的概率较低,故认为本方法适用于绝大多数氧化型染发产品中染发剂的测定。

### 2.5 提取溶剂的选择

依照《规范》^[25]^提供的样品前处理方法,取样品0.5 g,加入2 g/L亚硫酸氢钠水溶液-无水乙醇(1:1, v/v)至10 mL,超声提取15 min后对所得样品溶液进行色谱分析,发现对苯二胺、2-氨基-3-羟基吡啶、羟乙基对苯二胺等极性较大的染发剂出现“溶剂效应”,即色谱峰“分叉”。而用2 g/L亚硫酸氢钠水溶液将上述样品溶液稀释至25 mL后,可有效解决此问题。因此,将样品前处理方法改为取样0.5 g,加入2 g/L亚硫酸氢钠水溶液和无水乙醇混合溶液至10 mL,超声提取后再加2 g/L亚硫酸氢钠水溶液至25 mL。

进一步比较含10%、30%、50%、70%及90%体积分数乙醇的亚硫酸氢钠水溶液对四氨基嘧啶硫酸盐、2-氨基-3-羟基吡啶、对苯二胺、甲苯-2,5-二胺硫酸盐、间氨基苯酚、间苯二酚、2-甲基间苯二酚、4-氨基-2-羟基甲苯、4-氨基-3-硝基苯酚、2,6-二羟乙基氨甲苯、4-氯间苯二酚、1-萘酚等12个使用频率较高且极性差异大的染发剂的提取效果。经对比,认为染发剂整体提取效果随乙醇体积分数的增大而提升。但发现,采用含90%乙醇的亚硫酸氢钠水溶液制备样品溶液时,即使加入亚硫酸氢钠水溶液稀释,对苯二胺等成分仍存在溶剂效应,色谱峰峰形不佳;采用含70%乙醇的亚硫酸氢钠水溶液提取时,整体效果仅次于90%组。综上,选定含70%乙醇的亚硫酸氢钠水溶液作为提取溶剂,并确定1.3节的样品前处理方法。

### 2.6 方法学考察

2.6.1 标准曲线、检出限与定量限

在1.4节色谱条件下分析标准溶液,以染发剂的质量浓度(*C*, mg/L)为横坐标、峰面积(*A*)为纵坐标建立标准曲线,结果见表3。40种染发剂在各自范围内线性关系良好,相关系数(*r*)均大于0.9998。

**表3:** 40种染发剂的回归方程、线性范围、相关系数、检出限、定量限和加标回收率(*n*=3)

No.	Regression equation	Linear range/ (mg/L)	r	LOD/ (μg/g)	LOQ/ (μg/g)	2.5 mg/g			5.0 mg/g			10 mg/g		
						Recovery/%	RSD/%		Recovery/%	RSD/%		Recovery/%		RSD/%
1	A=3.863C-13.43	5-500	0.9999	81	272	100.8	1.8		98.8	1.1		105.3		0.7
2	A=9.655C-42.96	5-500	0.9999	50	167	98.7	0.8		101.2	0.6		99.6		0.3
3	A=7.604C-3.328	5-500	0.9999	50	167	109.6	2.6		105.2	0.8		102.4		0.6
4	A=22.08C+8.847	5-500	0.9999	50	168	101.8	0.6		103.5	0.5		100.5		0.9
5	A=8.821C-9.130	5-500	0.9999	48	160	103.5	1.8		98.6	0.8		105.1		0.7
6	A=16.24C+13.34	5-500	0.9999	25	84	95.5	3.6		94.2	1.0		93.8		1.4
7	A=17.30C+1.879	5-500	0.9999	25	84	102.6	3.1		99.8	0.5		97.8		0.8
8	A=21.40C+45.94	5-500	0.9999	10	33	103.0	2.3		102.3	0.4		100.1		0.9
9	A=4.581C+5.838	5-500	0.9999	50	167	98.3	0.5		101.0	0.9		99.2		0.4
10	A=9.828C+11.59	5-500	0.9999	25	83	100.7	1.5		101.8	0.4		98.6		0.9
11	A=9.842C+2.185	5-500	0.9999	25	83	98.7	0.9		99.9	0.6		98.5		0.9
12	A=17.08C+17.03	5-500	0.9999	10	34	105.1	3.9		99.4	0.6		97.4		0.9
13	A=12.66C+10.48	5-500	0.9998	25	84	101.9	1.0		87.0	2.0		86.5		1.9
14	A=16.16C+5.680	5-500	0.9999	10	33	106.0	2.3		100.0	0.5		98.8		0.8
15	A=4.156C-0.3880	5-500	0.9998	50	166	102.6	2.6		94.8	1.8		92.9		1.3
16	A=15.25C+12.51	5-500	0.9999	25	83	102.9	3.6		98.2	1.1		95.6		1.0
17	A=8.834C+6.080	5-500	0.9999	50	168	98.8	0.4		100.1	0.5		97.8		1.2
18	A=4.890C+0.5869	10-500	0.9999	168	504	100.5	0.8		103.6	1.7		101.7		1.2
19	A=4.119C+6.583	5-500	0.9999	84	279	95.4	2.3		92.6	2.4		90.4		1.5
20	A=15.97C-5.058	5-500	0.9999	50	165	101.6	3.5		96.5	1.0		93.3		0.8
21	A=9.363C+7.574	5-500	0.9999	25	84	102.1	4.0		97.4	1.4		95.7		1.1
22	A=15.52C+13.09	5-500	0.9999	25	83	104.3	1.9		98.0	1.2		96.1		0.5
23	A=13.42C+18.91	5-500	0.9999	25	82	93.5	1.1		88.9	3.2		101.8		0.2
24	A=29.03C+33.56	5-500	0.9999	10	34	96.3	2.1		104.2	1.0		105.9		0.6
25	A=13.36C+21.38	5-500	0.9999	25	84	96.4	3.5		95.8	1.4		93.8		1.2
26	A=27.47C+32.53	5-250	0.9999	5	17	97.6	1.0		102.3	0.4		99.5		0.9
27	A=27.26C+36.18	5-500	0.9999	10	33	96.3	0.3		93.0	2.8		89.9		2.2
28	A=13.43C+11.58	5-500	0.9999	25	83	95.6	0.4		98.2	0.8		96.1		1.0
29	A=15.09C+16.83	5-500	0.9999	50	165	99.0	1.9		99.7	0.9		93.3		0.5
30	A=7.479C+10.23	5-500	0.9999	50	166	94.5	1.6		95.9	1.8		101.2		0.7
31	A=8.930C+11.22	5-500	0.9999	50	166	100.2	1.8		103.2	1.9		106.2		1.0
32	A=13.91C+18.68	5-500	0.9999	50	165	92.6	0.4		92.5	0.3		96.5		0.5
33	A=25.45C+40.23	5-500	0.9999	10	34	95.0	0.2		97.1	0.9		100.9		0.3
34	A=7.039C+4.692	5-500	0.9999	25	84	94.9	1.1		100.0	0.7		99.5		0.6
35	A=5.679C+6.342	5-500	0.9999	25	83	90.4	0.9		91.7	1.1		92.9		1.4
36	A=6.771C+7.516	5-500	0.9999	10	34	96.0	0.8		97.8	1.0		102.5		2.4
37	A=2.318C+0.4190	5-500	0.9999	25	82	91.5	0.8		92.4	1.7		94.9		1.1
38	A=6.514C+8.196	5-500	0.9999	5	17	90.0	0.5		91.0	1.0		94.9		2.0
39	A=23.44C+43.40	5-250	0.9998	5	16	89.8	3.2		90.8	2.1		94.0		1.8
40	A=7.160C+1.060	5-500	0.9999	5	17	81.4	1.8		87.4	2.2		88.8		2.1

Nos. 1-40 were the same as that in Table 1. *A*: peak area; *C*: mass concentration, mg/L.

称取0.5 g(精确至0.001 g)空白基质样品(配方中不含本研究测定的40种染发剂),分别加入不同浓度的标准溶液,按1.3节方法进行前处理,分别以信噪比*S/N*为3和10时对应的含量作为检出限(LOD)与定量限(LOQ), 40种染发剂检出限为5~168 μg/g,定量限为16~504 μg/g,结果见表3。

2.6.2 加标回收率

取基质空白样品,每份0.5 g(精确至0.001 g),分别加入标准储备溶液1.25、2.5和5.0 mL(相当于样品中染发剂含量分别为2.5、5.0和10 mg/g),每个水平制备平行样品3份,按1.3节方法进行前处理,在1.4节色谱条件下进行测定。计算各成分平均回收率及其RSD,结果见表3。40种染发剂的平均回收率为81.4%~109.6%, RSD值均小于5%。

2.6.3 稳定性

取1.2节制备的100 mg/L标准溶液,按1.4节色谱条件分别于第0、4、8、12、16、20和24 h进行测定,计算各染发剂峰面积的RSD值。结果表明,40种染发剂在24 h内峰面积RSD值为0.2%~2.2%,说明稳定性良好。

### 2.7 实际样品测定

选取12批不同品牌、不同配方且添加染发剂种类较多的氧化型染发产品进行含量测定。12批样品中共检出24种准用染发剂,累计检出数量为66个,其在产品染发膏中的检出含量为0.01%~2.83%,全部符合《规范》^[25]^中的染发剂使用限值规定。所有样品均未检出对甲基氨基苯酚硫酸盐,1批样品中检出*N*,*N*-双(2-羟乙基)-对苯二胺硫酸盐,此染发剂具有单一紫外吸收光谱图谱,无对甲基氨基苯酚硫酸盐干扰,可利用本方法实现准确定量。

## 3 结论

本研究建立了高效液相色谱法测定氧化型染发类产品中40种染发剂,方法操作简便,准确性和稳定性良好,利用单一液相色谱系统进行分析,有效提高了实际应用中的检测效率;研究在《规范》检验方法基础上增加了13种准用染发剂,基本覆盖了氧化型染发产品配方中的常用染发剂。根据2016~2018年注册的染发产品配方统计结果,以及多个品牌产品的实际测定结果,认为本方法适用于绝大多数氧化型染发产品的检验检测。
